# Impact of prenatal exposure to delta 9‐tetrahydrocannabinol and cannabidiol on birth size and postnatal growth trajectories

**DOI:** 10.1111/ijpo.13187

**Published:** 2024-12-16

**Authors:** Brianna F. Moore, Noel T. Mueller, Wei Perng, Katherine A. Sauder, Emily T. Hébert, Adrienne T. Hoyt, Erica M. Wymore, Kristen E. Boyle, Emily J Su, Allison L. B. Shapiro, Gregory Kinney, Cristina Sempio, Jost Klawitter, Uwe Christians, Dana Dabelea

**Affiliations:** ^1^ Department of Epidemiology Colorado School of Public Health Aurora Colorado USA; ^2^ Lifecourse Epidemiology of Adiposity and Diabetes (LEAD) Center University of Colorado Anschutz Medical Campus Aurora Colorado USA; ^3^ Department of Pediatrics, School of Medicine University of Colorado Anschutz Medical Campus Aurora Colorado USA; ^4^ Department of Implementation Science Wake Forest University School of Medicine Winston‐Salem North Carolina USA; ^5^ Department of Health Promotion and Behavioral Sciences The University of Texas Health Science Center Austin Texas USA; ^6^ Department of Obstetrics and Gynecology School of Medicine, University of Colorado Anschutz Medical Campus Aurora Colorado USA; ^7^ Department of Anesthesiology University of Colorado School of Medicine Colorado Aurora USA

**Keywords:** adiposity, BMI, breastfeeding, cannabidiol, cannabis, delta‐9‐tetrahydrocannabinol, foetal growth restriction, growth trajectory, postnatal weight gain

## Abstract

**Background:**

Prenatal exposure to cannabis (or more specifically, delta 9‐tetrahydrocannabinol [Δ9‐THC]) has been consistently linked to low birthweight. Animal models further show that Δ9‐THC is associated with rapid postnatal growth. Whether this association is modified by breastfeeding is unknown.

**Methods:**

In this exploratory study, we followed 128 mother–child pairs through 3 years. Urinary Δ9‐THC and cannabidiol (CBD) were measured mid‐gestation. Generalized linear models estimated the associations between Δ9‐THC and neonatal body composition. A mixed‐effects model estimated the association between Δ9‐THC and body mass index (BMI) *z*‐score trajectories. Interaction was assessed by a three‐way product term (Δ9‐THC × breastmilk months × age).

**Results:**

Fifteen children (12%) had Δ9‐THC exposure; three had concomitant CBD exposure. Prenatal exposure to Δ9‐THC alone was associated with lower fat mass (−95 g, 95% confidence interval [CI]: −174, −14) and neonatal adiposity (−2.1%; 95% CI: −4.2, −0.4) followed by rapid postnatal growth (0.42 increase in BMI *z*‐score per square root year; 95% CI: 0.12, 0.72). Breastfeeding modified this association (*p* = 0.04), such that growth was similar for those breastfed for 5 months whereas a shorter duration of breastfeeding was associated with 1.1 higher BMI *z*‐score at 3 years (95% CI: 0.21, 2.05).

**Conclusions:**

Our study suggests that prenatal exposure to Δ9‐THC may alter early‐life growth. Breastfeeding may stabilize rapid postnatal growth, but the impact of lactational exposure requires further investigation.

## 1 INTRODUCTION

Over the past few decades, there has been a major shift in legality and attitudes towards cannabis use. As of 2024, cannabis is legal for recreational use in several countries (including Uruguay, Canada, South Africa, and Mexico) and 23 of 50 states in the United States. As cannabis becomes more socially acceptable, it is important to study the health effects, particularly in pregnancy.

Human epidemiologic studies have linked cannabis use during pregnancy with foetal growth restriction^1^, ^2^ and low birthweight.^3^, ^4^, ^5^, ^6^, ^7^, ^8^, ^9^, ^10^, ^11^ However, since these studies relied on self‐report, it is unclear whether a specific component of cannabis is driving this association. Cannabis contains many cannabinoids, with delta‐Δ9‐tetrahydrocannabinol (Δ9‐THC) and cannabidiol (CBD) being the most common. Both Δ9‐THC and CBD interact with the endocannabinoid system, a complex signaling system involved in metabolism and glucose homeostasis. Δ9‐THC directly interacts with cannabinoid 1 (CB1) and cannabinoid 2 (CB2) receptors.^12^ Conversely, CBD indirectly interacts with CB1 and CB2 receptors, and may counteract some of the effects of Δ9‐THC.^12^ As such, murine models report that Δ9‐THC exposure decreases birthweight,^13^, ^14^, ^15^, ^16^, ^17^ whereas CBD may have no impact on birthweight^18^ or may slightly increase it.^19^ However, whether Δ9‐THC and CBD act synergistically or antagonistically is unknown, as no human or animal studies have examined their individual or joint effects. This may have public health relevance, given the dramatic changes in the potency and ratio of Δ9‐THC:CBD in commercial cannabis products in recent years.^20^

Prenatal exposure to Δ9‐THC or CBD may also influence postnatal growth. Animal models show that prenatal exposure to Δ9‐THC is associated with rapid weight gain in infancy,^13^, ^14^, ^15^ whereas the effects of CBD on postnatal growth are less clear.^21^, ^22^ One epidemiologic study reported that co‐exposure to tobacco and cannabis was associated with a rapid increase in BMI trajectory from birth to middle childhood.^23^ However, the impact of prenatal exposure to Δ9‐THC on postnatal growth trajectories has yet to be examined in longitudinal cohorts.

Clinicians use growth trajectories to make recommendations for stabilizing child growth. One such recommendation is to encourage mothers to exclusively breastfeed for about 6 months.^24^ A longer duration of breastfeeding is associated with healthier growth in infancy^25^ and has been shown to protect against obesogenic effects of environmental exposures.^26^ However, these benefits may be negated by cannabinoid exposure via breast milk. Thus, it is critical to examine whether breastfeeding may impact the relationship between prenatal exposure to cannabis and postnatal growth.

To address the important research gaps, we assessed the associations between prenatal exposure to Δ9‐THC with birth size and postnatal growth trajectories through age 3 years. We hypothesized that prenatal exposure to Δ9‐THC, particularly in the absence of CBD, would be associated with lower neonatal adiposity followed by rapid growth in the first 3 years of life. We further hypothesized that a longer duration of exclusive breastfeeding would temper rapid postnatal growth among Δ9‐THC‐exposed offspring.

## 2 METHODS

We leveraged a racially and ethnically diverse Colorado‐based cohort: the Healthy Start study. Healthy Start began in 2010 as a study to better understand how overnutrition in pregnancy impacts obesity and cardiometabolic health of the offspring (NCT02273297). Pregnant women were recruited from the outpatient obstetrics clinics at the University of Colorado Hospital prior to 24 weeks of gestation. Women were excluded from this study if they were expecting multiple births or had pre‐existing diabetes, asthma, cancer, or psychiatric illness. Enrolled pregnant women were invited to participate in visits during pregnancy, at delivery, and after their child was born.

This exploratory study was conducted among a convenience subsample of 199 participants with available maternal urine collected at ~27 weeks gestation (the sample size was further reduced to 128 participants for the adiposity analysis and 140 participants for the trajectory analysis after exluding participants with missing data). The goal of this study was to investigate the impact of prenatal exposure to cannabis on neonatal body composition and BMI growth trajectories through 3 years of age.

### 2.1 Adiposity measures

At the delivery visit, neonatal fat mass and fat‐free mass were measured via whole body air displacement plethysmography (PEA POD, COSMED, Rome, Italy) within 72 h of delivery. The PEA POD system employs densitometric techniques to measure total body mass and two compartments in the offspring: fat mass (adipose tissue) and fat‐free mass. Neonatal adiposity (fat mass percentage) was calculated as a proportion of the fat mass divided by total mass. Fat mass and fat‐free mass were conducted twice. A third exam was conducted if fat mass percentage differed by >2%. The mean of the two closest measures was used in analyses.

### 2.2 Growth measures

Childhood weight, recumbent length (generally until 24 months), and standing height (generally after 24 months) were abstracted from medical records. These measurements were generally recorded at well‐child visits, which are recommended at 1, 2, 4, 6, 12, 18, 24, 30, and 36 months of age. BMI was calculated by dividing weight in kilograms by height in meters squared.

### 2.3 Prenatal exposure to cannabinoids

We measured Δ9‐THC, CBD, and its major metabolites in maternal urine collected at ~27 weeks gestation. Samples were analysed by iC42 Clinical Research and Development (Aurora, CO, USA), using a validated, specific, and highly sensitive liquid chromatography–tandem mass spectrometry (LC–MS/MS) assay.^27^ The LC–MS/MS system consisted of a 1200 high‐performance liquid chromatography system (Agilent Technologies, Palo Alto, CA, USA) and an API5000 tandem mass spectrometer (Sciex, Concord, ON, Canada) connected via an atmospheric pressure chemical ionization source run in the positive mode. The lower limits of quantification (LOQs) ranged from 0.39 ng/mL (for Δ9‐THC) to 7.82 ng/mL (for Δ9‐THC‐C‐gluc) (see Table 2).

Prenatal exposure to Δ9‐THC, alone or in combination with CBD, was categorized two ways. First, we characterized exposure to Δ9‐THC as not exposed (Δ9‐THC and its metabolites were below the LOD) and exposed (Δ9‐THC or metabolites exceeded the LOD). Second, we characterized exposure to Δ9‐THC alone as: not exposed (Δ9‐THC and/or its metabolites were not detected in the absence of CBD or its metabolites) and exposed (Δ9‐THC or its metabolites were detected, but CBD or its metabolites were not detected).

### 2.4 Covariates

Data on maternal education, household income, race, and ethnicity were collected through research questionnaires. Maternal age at delivery was calculated from delivery date and maternal date of birth. Maternal height was measured using a stadiometer at the first research visit. Pre‐pregnancy weight was obtained from medical records or from questionnaires completed at enrolment. Gestational weight gain was calculated as the difference between the last available weight measurement during pregnancy and the pre‐pregnancy weight. Maternal psychiatric disorders (non‐specified) and gestational diabetes status were obtained from medical records. Cotinine was measured in urine samples collected at 27 weeks gestation via solid‐phase competitive ELISA (Calbiotech Inc., El Cajon, CA, USA), with a sensitivity of 1 ng/mL. Prenatal exposure to tobacco was dichotomized as exposed (cotinine ≥ the limit of detection [LOD]; 0.05 ng/mL) and not exposed (cotinine < LOD). Mothers were asked to report the number of adults in the household (including themselves) who were regular smokers when their child was 5 months of age. Responses to this question ranged from 0 to 6. Self‐report of household cigarette smokers was dichotomized as no household smokers or any household smokers. Breastfeeding for at least 5 months was ascertained by asking mothers whether they were currently breastfeeding at the 5‐month visit.

### 2.5 Statistical analysis

Generalized linear models were used to estimate the association between prenatal exposure to Δ9‐THC and birthweight, fat mass, fat‐free mass, and adiposity. Covariates were selected by using a directed acyclic diagram. Our base models adjusted for maternal age, household income, gravidity, gestational age at urine collection, gestational diabetes, maternal psychiatric disorder, pre‐pregnancy BMI, gestational weight gain, offspring sex, gestational age at birth, and child race and ethnicity. We additionally adjusted for prenatal exposure to tobacco because co‐use of tobacco and cannabis is common^28^ and tobacco is independently associated with child growth.^29^ We further included birth length as a precision variable, given that neonatal body composition may vary by birth length. We present adjusted beta coefficients and means with corresponding 95% confidence intervals (CIs) for all models.

A mixed‐effects regression model examined the longitudinal association between prenatal exposure to Δ9‐THC with postnatal growth trajectories (1–36 months). We modeled both BMI trajectories and BMI *z*‐score trajectories to leverage the advantages of each approach. BMI *z*‐score trajectories allow for comparison of the child's BMI relative to peers over time. This is particularly useful for paediatric populations, because boys and girls follow different growth trajectories.^30^ On the other hand, BMI trajectories describe the child's BMI change over time^31^ while also allowing for a simpler interpretation. Based on the deviance information criteria, a square root transformation of age yielded the best fitting trajectory. We used Wald tests with Kenward–Roger degrees of freedom and an unstructured covariance matrix. To test for interaction by breastfeeding, we introduced a three‐way product term (Δ9‐THC × breastfeeding × the square root of age) and lower order product terms in a mixed‐effects regression model. The mixed‐effects regression models adjusted for all covariates listed above, as well as self‐report of household smokers at age 5 months.

All statistical analyses were conducted using Stata, Version 14.2 (StataCorp LP, College Station, TX, USA). An alpha level of 0.05 was used to determine statistical significance for all analyses, including interactions.

## 3 RESULTS

Of the 1410 participants initially enrolled in Healthy Start, 199 participants had available urine samples for Δ9‐THC/CBD analysis. The adiposity analytic sample was 128 participants, after excluding 49 participants who did not undergo the PEA POD assessment and 22 participants with missing covariate data. The trajectory analytic sample was 140 participants, after excluding 8 with insufficient length/height and weight measurements and 51 participants with missing covariate data. As compared to the overall cohort (*n* = 1410), mothers in the adiposity analytic sample (*n* = 128) were slightly older, had higher incomes, and were less likely to have prenatal exposure to tobacco (Table 1).

**TABLE 1:** Characteristics of eligible mothers and children in the Healthy Start study.

		Prenatal exposure to Δ9‐THC^a^		
	All (*n* = 128)	Not exposed (*n* = 113)	Exposed (*n* = 15)	*p*‐value
Mother characteristics				
Age (years)	30 ± 6	30.3 ± 5.5	26.3 ± 5.4	*p* < 0.01
Gravidity	1.4 ± 1.4	1.4 ± 1.4	1.5 ± 1.5	*p* = 0.80
Pre‐pregnancy BMI (kg/m^2^)	24.8 ± 4.6	24.8 ± 4.7	24.8 ± 4.4	*p* = 0.98
Gestational weight gain (kg)	14.8 ± 5.2	14.3 ± 4.9	18.1 ± 6.2	p < 0.01
Gestational diabetes (yes/borderline)	17 (13%)	15 (13%)	2 (13%)	*p* = 0.96
Household income ≥$70 000	56 (44%)	53 (47%)	3 (20%)	*p* = 0.11
Some college education	98 (77%)	91 (81%)	7 (47%)	*p* = 0.01
Prenatal exposure to tobacco (maternal cotinine ≥ LOD)	19 (15%)	9 (8%)	10 (67%)	p < 0.01
Gestational age at urine collection (weeks)	26.5 ± 2.7	26.6 ± 2.7	25.8 ± 2.9	*p* = 0.71
Race and ethnicity				
Hispanic	33 (26%)	31 (27%)	2 (13%)	
Non‐Hispanic black	13 (10%)	7 (6%)	6 (40%)	
Asian	11 (9%)	9 (8%)	2 (13%)	
Pacific Islander				
American Indian or Alaska Native				
Non‐Hispanic white	71 (56%)	66 (58%)	5 (33%)	p < 0.01
Child characteristics				
Female	73 (57%)	62 (55%)	11 (73%)	*p* = 0.18
Gestational age at birth (weeks)	39.5 ± 1.2	39.5 ± 1.2	39.4 ± 1.2	*p* = 0.76
Breastfeeding at age 5 months (yes)				
Household smokers at 5 months (any)	12 (10%)	9 (8%)	3 (21%)	*p* = 0.13

*Note*: Continuous variables are expressed as means ± standard deviation. Independent samples *t*‐tests were used to examine the differences in means by cannabis categories. Categorical variables are expressed as proportions of column totals. Chi‐square tests were used to examine differences in proportions by cannabis categories.

Abbreviations: Δ9‐THC, delta 9‐tetrahydrocannabinol; BMI, body mass index; LOD, limit of detection.

^a^ The categories of were as follows: not exposed (Δ9‐THC below the LOD) and exposed (Δ9‐THC exceeded the LOD).

Within the adiposity analytic sample (*n* = 128), over half (51%) of the children were identified as non‐Hispanic White, 31% identified as Hispanic, 9% identified as non‐Hispanic Black, 2% identified as Asian, 2% identified as American Indian or Alaska native, and 6% identified as being multiracial. Most of the women had some college education (77%) and many had a household income ≥$70 000 (44%).

In the adiposity analytic sample (*n* = 128), 12% (*n* = 15) had detectable Δ9‐THC metabolites at ~27 weeks gestation (Table 2). Of these, three participants had concomitant CBD exposure. Pregnant people with Δ9‐THC exposure tended to be younger with less education and higher gestational weight gain. Δ9‐THC‐exposed children were breastfed for a shorter duration, were more likely to have prenatal and postnatal exposures to tobacco and were more likely to identify as Hispanic or non‐Hispanic White (Table 1). There were no differences in gravidity, pre‐pregnancy BMI, gestational diabetes, gestational age at urine collection, gestational age at birth, or offspring sex.

**TABLE 2:** Summary of urinary cannabinoids and cannabinoid metabolites^a^ measured in maternal urine collected at ~27 weeks gestation, *n* = 128.

Compound	Δ9‐THC	Δ9‐THC‐gluc	Δ9‐THC‐COOH	Δ9‐THC‐C‐gluc	CBD
Limit of quantification (ng/mL)	0.39	1.56	0.78	7.82	0.78
Limit of detection (ng/mL)	0.20	0.78	0.39	3.91	0.39
Minimum (ng/mL)	0.3	0.8	0.4	4.5	0.5
Maximum (ng/mL)	0.9	24.1	20.1	1091.3	0.8
Number of positive	5	7	12	10	3
Number of negative	123	121	116	118	125

Abbreviations: Δ9‐THC, delta 9‐tetrahydrocannabinol; BMI, body mass index; CBD, cannabidiol.

^a^ No positive results for 11OH‐Δ9‐THC, CBD‐gluc, 6α‐OH‐CBD, 6β‐OH‐CBD, 7‐OH‐CBD, and CBD‐COOH.

Neonates with prenatal exposure to Δ9‐THC alone had lower fat mass (−95 g; 95% CI: −174, −14; *p* = 0.05) and adiposity (−2.1%; 95% CI: −4.2, −0.04, *p* = 0.04), as compared to non‐exposed neonates (Table 3). However, these associations were slightly attenuated following adjustment for prenatal exposure to tobacco (Model 3) and further attenuated following adjustment for birth length (Model 4). There was no difference in birthweight or neonatal fat‐free mass according to prenatal exposure to Δ9‐THC.

**TABLE 3:** Adjusted^a^ means and mean differences for the associations between prenatal exposure to Δ9‐THC and neonatal body composition, *n* = 128.

			Adjusted means and beta coefficients		
Δ9‐THC categories	*n*	Birth weight (g)	Fat‐free mass (g)	Fat mass (g)	Adiposity (% fat mass)
Model 1: Δ9‐THC and CBD^b^					
Not exposed	113	3333 (3264, 3401)	2905 (2859, 2953)	317 (288, 346)	9.5% (8.8, 10.3)
Exposed	15	−160 (−376, 56), *p* = 0.15	−110 (−262, 41), *p* = 0.15	−73 (−145, 0), *p* = 0.05	−1.5 (−3.5, 0.3), *p* = 0.09
Model 2: Δ9‐THC alone^c^					
Not exposed	116	3343 (3267, 3403)	2895 (2847, 2945)	317 (289, 346)	9.8% (8.9, 10.6)
Exposed	12	−216 (−458, 27), *p* = 0.08	−157 (−331, 17), *p* = 0.08	−95 (−174, −14), *p* = 0.02	−2.1 (−4.2, −0.04), *p* = 0.04
Model 3: Δ9‐THC alone^d^					
Not exposed	116	3331 (3263, 3399)	2891 (2843, 2938)	317 (287, 347)	9.6% (8.8, 10.3)
Exposed	12	−176 (−445, 92), *p* = 0.20	−101 (−283, 81), *p* = 0.28	−68 (−152, 16), *p* = 0.11	−2.3 (−4.8, 0.03), *p* = 0.08
Model 4: Δ9‐THC alone^e^					
Not exposed	116	3320 (3257, 3384)	2882 (2839, 2924)	314 (286, 342)	9.5% (8.7, 10.2)
Exposed	12	−62 (−339, 216), *p* = 0.66	−4 (−194, 185), *p* = 0.96	−44 (−132, 44), *p* = 0.33	−1.3 (−3.9, 1.2), *p* = 0.31

Abbreviations: Δ9‐THC, delta 9‐tetrahydrocannabinol; CBD, cannabidiol; CI, confidence interval; LOD, limit of detection.

^a^ Models 1 and 2 adjusted for maternal age (years), household income (≥$70 000, <$70 000 or missing/declined to answer), maternal education (High school degree or less, some college), maternal race/ethnicity (Hispanic, non‐Hispanic black, non‐Hispanic other, and non‐Hispanic white), gravidity, gestational diabetes (no, borderline/yes), age at urine collection (weeks), pre‐pregnancy body mass index (kg/m^2^), gestational weight gain (kg), offspring sex, and gestational age at birth (weeks).

^b^ The categories were as follows: not exposed (Δ9‐THC and its metabolites below the LOD) and exposed (Δ9‐THC and/or any of its metabolites exceeded the LOD).

^c^ The categories were as follows: not exposed (Δ9‐THC was not detected in the absence of CBD) and exposed (Δ9‐THC alone was detected, without CBD).

^d^ Model 3 adjusted for prenatal exposure to tobacco (cotinine measured in maternal urine at 27 weeks gestation<LOD, cotinine>LOD), in addition to all other variables in Model 2.

^e^ Model 4 adjusted for birth length (cm), in addition to all other variables in Model 3.

**FIGURE 1:**
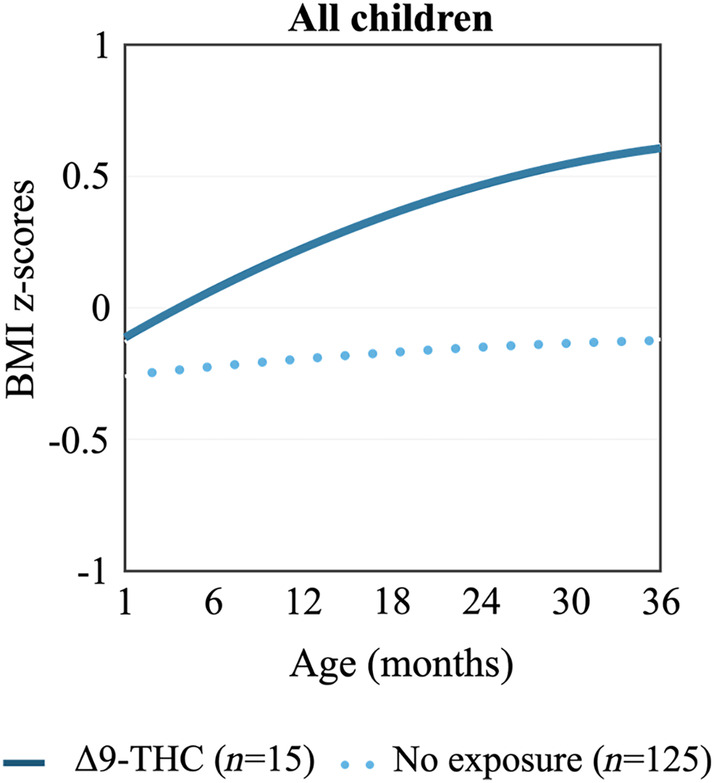
BMI *z*‐score trajectories among children with prenatal exposure to Δ9‐THC (*n* = 15) and children with no prenatal exposure to Δ9‐THC (*n* = 125). The rate of growth in BMI *z*‐score was more rapid among Δ9‐THC‐exposed offspring, as compared to unexposed offspring (0.42 per square root year; 95% CI: 0.12, 0.72; *p* < 0.01). Δ9‐THC, delta 9‐tetrahydrocannabinol; BMI, body mass index; CI, confidence interval.

Compared to children with no exposure, children with prenatal exposure to Δ9‐THC experienced a more rapid BMI *z*‐score trajectory (Figure 1; 0.42 increase in BMI *z*‐score per year; 95% CI: 0.12, 0.72). This association was independent of prenatal or postnatal exposure to tobacco. A similar but non‐significant pattern was observed for BMI trajectories (Figure S1).

**FIGURE 2:**
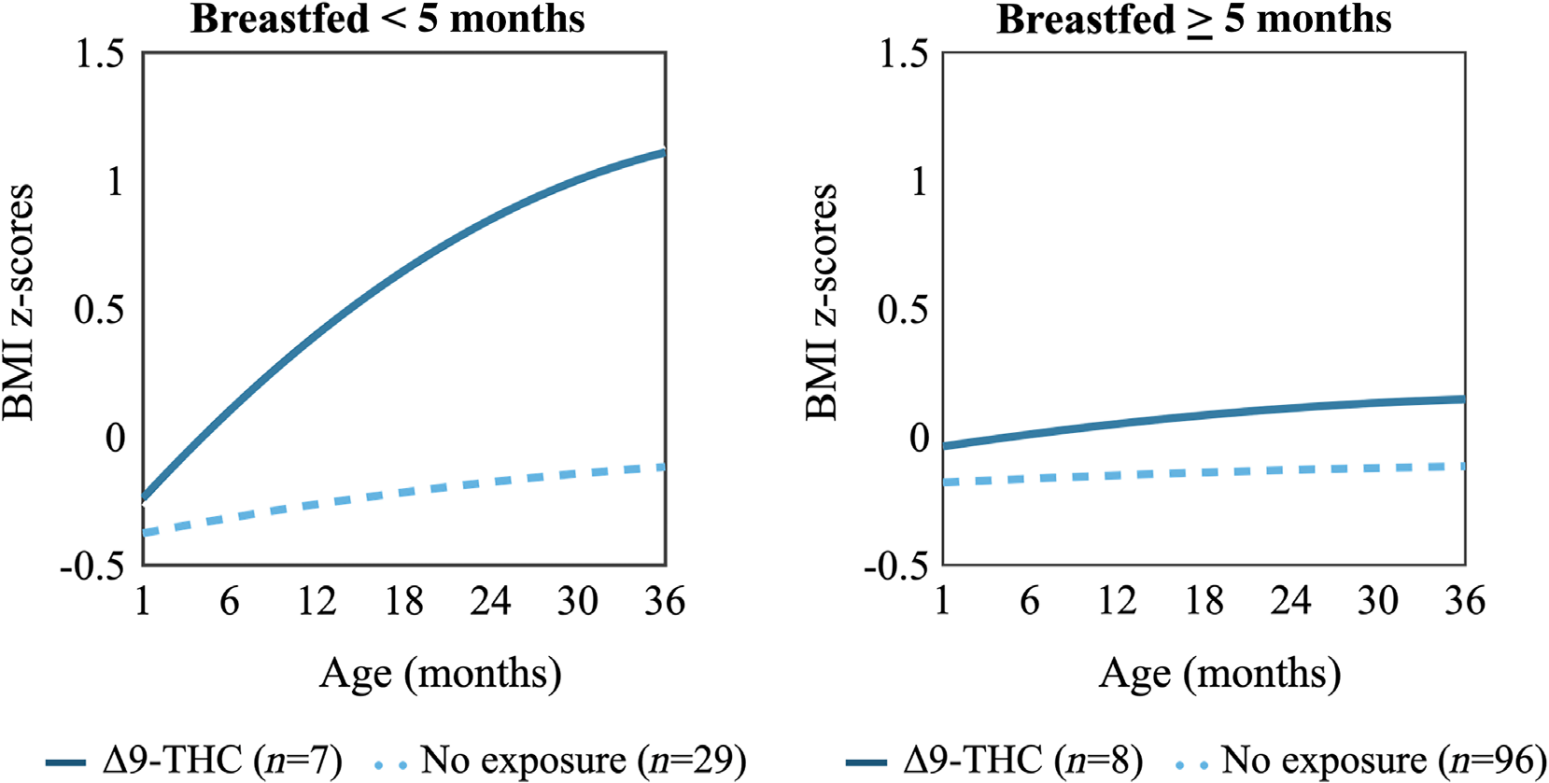
BMI *z*‐score trajectories according to prenatal exposure to Δ9‐THC and breastfeeding. There was evidence of effect modification by breastfeeding (*p*‐value for Δ9‐THC × breastfed × age interaction = 0.04), such that Δ9‐THC did not appear to influence growth among those breastfed for 5 months whereas a shorter duration of breastfeeding was associated with 1.1 higher BMI *z*‐score at 36 months (95% CI: 0.21, 2.05). Δ9‐THC, delta 9‐tetrahydrocannabinol; BMI, body mass index; CI, confidence interval.

Breastfeeding modified the association between prenatal exposure to Δ9‐THC and postnatal BMI *z*‐score trajectories (Figure 2; *p* for three‐way interaction = 0.04). For example, Δ9‐THC did not appear to influence growth among those breastfed for 5 months whereas a shorter duration of breastfeeding was associated with 1.1 higher BMI *z*‐score at 36 months (95% CI: 0.21, 2.05).

Similarly, breastfeeding modified the association between Δ9‐THC and postnatal BMI trajectories, but the interaction *p*‐value was not statistically significant (Figure S2).

## 4 DISCUSSION

Our results suggest that prenatal exposure to Δ9‐THC, particularly in the absence of CBD, is associated with a smaller size at birth followed by rapid postnatal growth. Our findings further suggest that the association between prenatal exposure to Δ9‐THC and postnatal growth trajectories is modified by breastfeeding, such that the longer the offspring was breastfed, the less prenatal exposure to Δ9‐THC impacted postnatal growth.

Prenatal exposure to Δ9‐THC, in the absence of CBD, influenced birth size. We speculate that this may be due to the distinct effects of the cannabinoids. Consistent with our pilot study, animal models have demonstrated that prenatal exposure to Δ9‐THC is associated with lower birthweight followed by rapid postnatal growth.^13^, ^14^ Conversely, the effect of CBD on offspring growth is not clear, with animal models showing little to no impact on birthweight^18^, ^19^ or its effects on postnatal growth appear to be sex‐specific.^21^, ^22^ The biological effects of Δ9‐THC and CBD on fetal growth may involve activation of CB1 and CB2 receptors, which influences brain, pancreas, and adipose tissue development of the fetus. Since Δ9‐THC has a higher affinity for CB1 and CB2 receptors than CBD, this could explain the seemingly stronger effects of Δ9‐THC on fetal growth we observed in this study. However, new evidence has emerged to suggest that Δ9‐THC and CBD interact with other non‐canonical receptors, such as inhibiting placental growth factor (PlGF) and vascular endothelial growth factor (VEGF) signaling pathways^32^, ^33^ (contributing to insufficient placental angiogenesis, a known driver of fetal growth restriction).^34^ While our study is a first step in understanding the independent effects of Δ9‐THC and CBD, considerable research is needed to address the paucity of data surrounding prenatal exposure to CBD, in the absence of Δ9‐THC, on child growth.

Our study results further suggest that Δ9‐THC‐exposed offspring grew more rapidly in early life. While some catch up growth is expected, over‐compensatory growth is generally a result of intrauterine growth restriction and increases the risk of metabolic diseases later in life.^35^ Findings from this study further suggest that a longer duration of breastfeeding may serve as a potential strategy for the normalizing growth among cannabis‐exposed offspring. Breastfeeding has numerous well‐documented benefits for both mother and infant. Breastfeeding may improve satiety, reduce problematic feeding behaviours, and/or provide cannabis‐exposed offspring with bioactive factors that have the potential to regulate growth.^36^ As such, any duration of exclusive breastfeeding tends to lower the future risk for obesity.^37^ However, these effects may be negated if the lactating person uses cannabis. Δ9‐THC accumulates in breastmilk and may be present up to 6 weeks following use.^38^, ^39^ Furthermore, cannabinoids may alter lipid profiles of breastmilk.^40^ While our results suggest that a longer duration of breastfeeding may protect against rapid postnatal growth, we did not specifically measure cannabinoids in breastmilk, as breastmilk was not collected in our study. Therefore, future studies are needed to quantify exposure via breastmilk and to determine the extent to which lactational exposure to Δ9‐THC impacts growth.

Tobacco is commonly used with cannabis ^28^ which makes it difficult to disentangle the effects of each exposure on child growth. While most studies report that the associations between prenatal exposure to cannabis and low birthweight are independent of tobacco,^1^, ^3^, ^4^, ^5^, ^6^, ^8^ some studies report null associations after adjustment for tobacco.^7^, ^9^ In our regression models, the associations between prenatal exposure to cannabis and birth size were attenuated following adjustment for tobacco. This may be due to tobacco explaining some of the same variance (as evidenced by the effect estimates moving towards the null) or a reduction in model precision (as evidenced by the widening of the confidence intervals). By contrast, in our mixed‐effects models, adjustment for prenatal and postnatal exposure to tobacco did not impact the results. This may suggest that prenatal exposure to tobacco may be less impactful on BMI trajectories as children age. Tobacco may also be an effect modifier. However, we lacked the sample size to explore the interaction between tobacco and cannabis. Prospective cohorts with sufficiently large subgroups of children with objective assessment of both cannabis and tobacco exposure during pregnancy are needed to understand the impact of polysubstance use on child growth.

Our study has some limitations. First, our study is limited by the cannabis exposure assessment. First, cannabinoids were measured only once at 27 weeks gestation. The urinary half‐life of Δ9‐THC‐metabolites is relatively short, ranging from 20 h (for light, infrequent users) to 10 days (for heavy, frequent use).^41^ Thus, our one‐time measure may fail to capture exposures early in pregnancy (when many people are just learning of their pregnancies) or during late gestation (when the majority of offspring adipose tissue growth occurs^42^) but more likely to capture heavy, frequent use. If the exposure misclassification were differential with respect to our outcome of interest, this may have biased the results away from the null. Second, cannabinoid concentrations may vary by urinary dilution between people. This is often overcome by correcting for urinary creatinine. However, creatinine was not measured as part of this study. Nevertheless, we believe that this represents a minor limitation to our approach since the goal of our study was to compare offspring with and without Δ9‐THC exposure rather than quantify a dose–response relationship. Finally, as described, we did not capture postnatal exposure, thus it is unclear if there are developmental windows when the child is more susceptible to cannabis.

Second, we lacked information on some important confounders. For instance, the use of alcohol and other substances during pregnancy have been linked to foetal growth restriction and may be used along with cannabis.^43^ Not adjusting for these confounders may have biased the results away from the null. Furthermore, although we adjusted for many relevant covariates, there is potential for residual confounding. In fact, it has been suggested that the associations between prenatal exposure to cannabis on offspring health outcomes may be explained by shared family‐based social and lifestyle factors, rather than by direct fetal programming.^44^ However, given the consistency of animal models^13^, ^14^, ^15^, ^16^, ^17^ and epidemiologic studies,^1^, ^2^, ^3^, ^4^, ^5^, ^6^, ^7^, ^8^, ^9^, ^10^ we conclude that prenatal exposure to Δ9‐THC is likely to be an important risk factor for altered prenatal and postnatal growth of the offspring.

Finally, our approach is limited by the small sample size and potential for selection bias for those who completed the follow‐up visits. Our analytic sample included mothers who were slightly older, had higher household incomes, were less likely to smoke cigarettes, and were more likely to have female offspring.

## 5 CONCLUSIONS

Over the past few decades, there has been a major shift in legality and attitudes towards cannabis use during pregnancy. Concurrently, there has also been a dramatic increase in the potency of cannabis.^20^ These trends are worrisome, as evidence suggests that cannabis use in pregnancy may contribute to adverse health effects in the offspring. Here, we found that prenatal exposure to Δ9‐THC, without evidence of CBD exposure, was associated with fetal growth restriction followed by rapid growth in early life. These effect estimates were non‐trivial. Δ9‐THC exposure was associated with a 216‐g reduction in birthweight and a 2.1% reduction in neonatal adiposity, which is comparable to the effect sizes of other known risk factors for low birthweight, such as malnutrition^45^ or smoking^46^ during pregnancy. A longer duration of exclusive breastfeeding may stabilize rapid growth among Δ9‐THC‐exposed offspring, but the impact of lactational exposure to Δ9‐THC requires further investigation. Considering the increasing use and acceptance of cannabis, these findings highlight the need for public health messaging surrounding cannabis use during pregnancy and while breastfeeding.

## AUTHOR CONTRIBUTIONS

B.F.M. initiated the cannabinoid analysis for this study, analysed the data, and wrote each draft of the manuscript. C.S., J.K., and U.C. performed the cannabinoid analyses and made substantial contributions to the interpretation of the cannabinoid exposure assessment. provided substantial feedback on the interpretation of these measures. N.T.M., W.P., K.A.S., E.T.H., A.T.H., E.M.W., K.E.B., E.J.S., A.L.B.S., G.K., and D.D. provided critical feedback on the clinical and mechanistic interpretation of the findings.

## FUNDING INFORMATION

This work was supported by the National Institutes of Health (R01DK076648, UH3OD023248, R01ES022934, R00ES028711, UL1TR003167, R21DA061104).

## CONFLICT OF INTEREST STATEMENT

The authors declare no conflicts of interest.

## CONSENT TO PARTICIPATE

Informed consent was obtained prior to participation in the parent study, which includes analysis of stored biospecimens.

## Supporting information


**Data S1.** Supporting Information.

## Data Availability

Data from the Healthy Start study are not publicly available because it contains sensitive personal health information. Deidentified data from the Healthy Start study are available upon reasonable request.
