# Supplementary material for: Impact of prenatal exposure to delta 9‐tetrahydrocannabinol and cannabidiol on birth size and postnatal growth trajectories
Source: Pediatr Obes. 2024 Dec 16;20(1):e13187. doi: 10.1111/ijpo.13187 (PMC11669837; doi:10.1111/ijpo.13187)
Supplement: Supplementary file 1 — Data S1. Supporting Information. [file IJPO-20-e13187-s001.pdf]

Supplemental Materials

Impact of prenatal exposure to delta 9-tetrahydrocannabinol and cannabidiol on birth size and postnatal growth trajectories: A pilot study

Authors: Brianna F. Moore,[1,2] Noel T. Mueller,[1,2,3] Wei Perng,[1,2] Katherine A. Sauder,[4] Emily T. Hébert,[5] Adrienne T. Hoyt,[5] Erica M. Wymore,[3] Kristen E. Boyle,[2,3] Allison L. B. Shapiro,[2,3] Gregory Kinney,[1] Cristina Sempio,[6] Jost Klawitter,[6] Uwe Christians,[6] Dana Dabelea[1,2,3]

Affiliations:

[1]Department of Epidemiology, Colorado School of Public Health, Aurora CO, USA.

[2]Lifecourse Epidemiology of Adiposity and Diabetes (LEAD) Center, University of Colorado Anschutz Medical Campus, Aurora CO, USA.

[3]Department of Pediatrics, School of Medicine, University of Colorado Anschutz Medical Campus, Aurora, CO, USA.

[4]Department of Implementation Science, Wake Forest University School of Medicine, Winston-Salem, NC, USA.

[5]Department of Health Promotion and Behavioral Sciences, The University of Texas Health Science Center, Austin TX, USA.

[6]Department of Anesthesiology, University of Colorado School of Medicine, Aurora, Colorado

Address correspondence to: Brianna Moore, Lifecourse Epidemiology of Adiposity and Diabetes (LEAD) Center, Colorado School of Public Health, Aurora CO, USA.

brianna.f.moore@cuanschutz.edu.

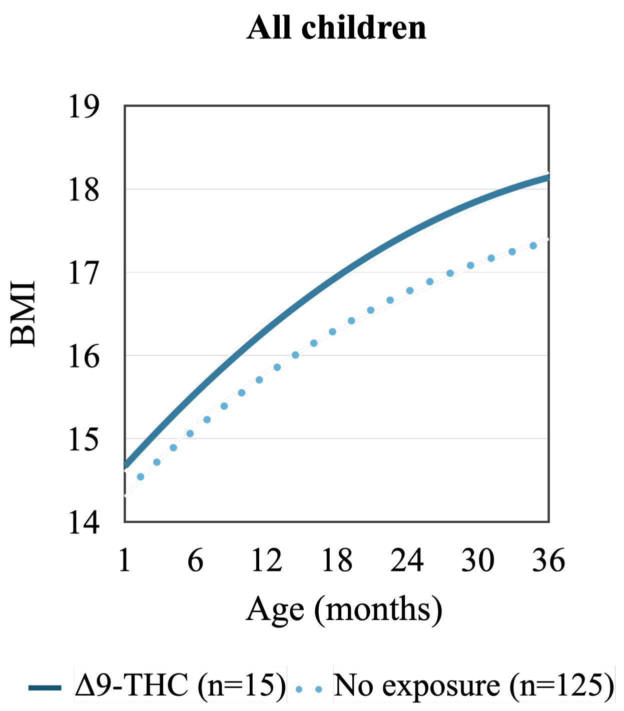

Supplemental Figure S1. BMI trajectories among children with prenatal exposure to Δ9-THC (n=15) and children with no prenatal exposure to Δ9-THC (n=125). The rate of growth in BMI was more rapid among Δ9-THC-exposed offspring, as compared to unexposed offspring (0.40 per square root year; 95% CI: -0.12, 0.92; p=0.13).

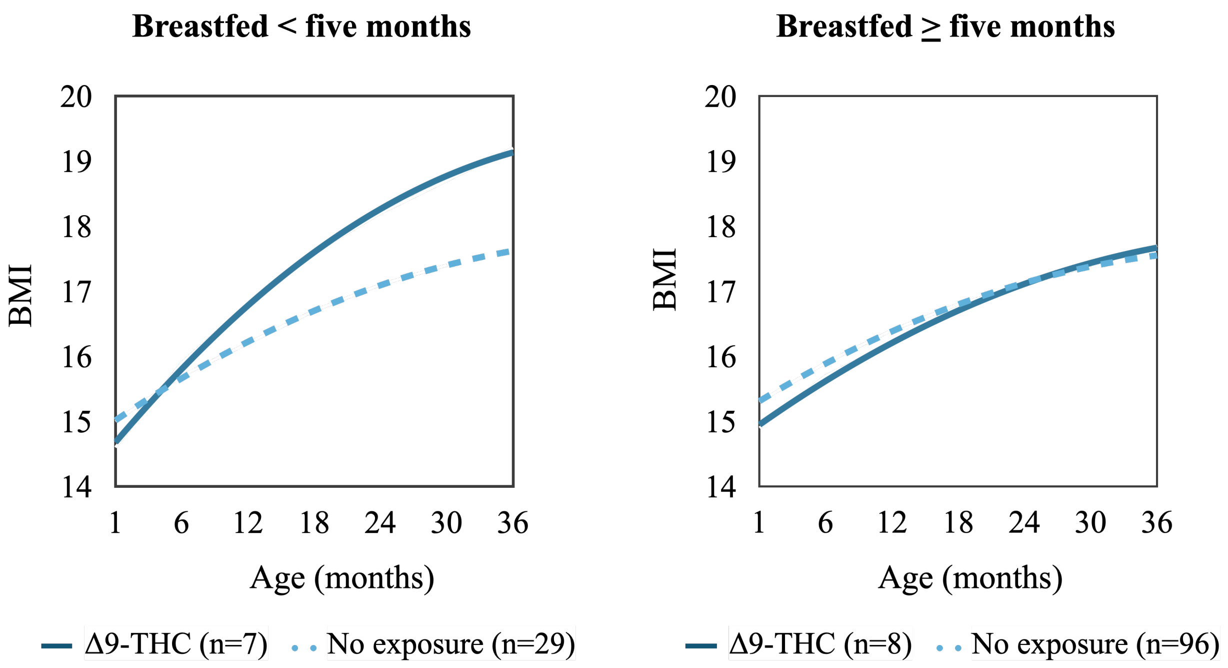

Supplemental Figure S2. BMI trajectories according to prenatal exposure to Δ9-THC and breastfeeding. There was evidence of effect modification by breastfeeding (p-value for Δ9-THC x breastfed x age interaction=0.12), such that Δ9-THC did not appear to influence growth among those breastfed for five months whereas a shorter duration of breastfeeding was associated with 1.8 higher BMI at 36 months (95% CI: 0.2, 3.2).
